# Oral findings in children on liver transplantation programming: a scoping review

**DOI:** 10.1590/1984-0462/2023/41/2022095

**Published:** 2023-05-15

**Authors:** Catielma Nascimento Santos, Claudia Jacqueline Lopez Gallegos, Raquel D’Aquino Garcia Caminha, Gilda Porta, Luiz Alberto Valente Soares, Marcelo Bönecker, Paulo Sérgio da Silva Santos

**Affiliations:** a Universidade de São Paulo, São Paulo, SP, Brazil.; b Universidade Federal de Sergipe, Lagarto, SE, Brazil.; c Universidade de São Paulo, Bauru, SP, Brazil.

**Keywords:** Liver disease, Oral manifestations, Child, Dentistry, Gastroenterology, Doença hepática, Manifestações bucais, Criança, Odontologia, Gastroenterologia

## Abstract

**Objective:**

To identify oral characteristics found in children with liver disease in programming for liver transplantation.

**Data source:**

The methodology was written according to PRISMA-ScR. We adopted the methodological framework and recommendations for this type of review by Arksey and O’Malley and the Joanna Briggs Institute. The protocol was registered in the Open Science Framework (https://doi.org/10.17605/OSF.IO/QCU4W). A systematic search (Medline/PubMed, Scopus, Web of Science, and ProQuest) was conducted to identify studies that met the inclusion criteria: systematic reviews; prospective clinical trials (parallel or crossover group designs); observational studies (cohort, case-control, and cross-sectional studies); clinical case series; and case reports evaluating children with liver disease in preparation for transplantation. The last search was conducted in July 2021, and no restrictions were imposed as to language or year of publication. Studies presenting mixed data with post-transplant evaluation, and studies evaluating not only liver transplantation but also other solid organs were excluded. Screening, inclusion, and data extraction were performed by two reviewers independently. A narrative synthesis was conducted to describe the findings of the study.

**Data synthesis:**

The bibliographic search identified 830 references. A total of 21 articles were read in their entirety after the inclusion criteria assessment. Finally, after evaluating the exclusion criteria, only 3 studies were considered for the qualitative analysis.

**Conclusions:**

Children with liver disease in preparation for transplantation may present enamel defects, tooth pigmentation, caries, gingivitis, and opportunistic infections such as candidiasis.

## RESUMO

**Objetivo:**

Identificar características bucais em crianças hepatopatas em programação para o transplante hepático.

**Fontes de dados:**

A metodologia foi descrita de acordo com o PRISMA-ScR. Adotamos a estrutura metodológica e recomendações para este tipo de revisão por Arksey e O’Malley e o Instituto Joanna Briggs. O protocolo foi registrado no Open Science Framework (https://doi.org/10.17605/OSF.IO/QCU4W). Uma pesquisa sistemática (Medline/PubMed, Scopus, Web of Science e ProQuest) foi conduzida para identificar estudos que preenchessem os critérios de inclusão: revisões sistemáticas; ensaios clínicos prospectivos (desenhos de grupos paralelos ou cruzados); estudos observacionais (coorte, caso-controle e estudos transversais); séries de casos clínicos; e relatos de casos que avaliam crianças com doenças hepáticas em preparação para o transplante. A última busca foi conduzida em julho de 2021, e não foram impostas restrições quanto ao idioma ou ano de publicação. Foram excluídos estudos que apresentavam dados mistos com avaliação pós-transplante e estudos que avaliavam não só o transplante de fígado, mas também de outros órgãos sólidos. O rastreio, inclusão e extração de dados foram realizados por dois revisores independentemente. Foi conduzida uma síntese narrativa para identificar os resultados do estudo.

**Síntese dos dados:**

A pesquisa bibliográfica identificou 830 referências. Foram lidos 21 artigos na íntegra após avaliação dos critérios de inclusão. Finalmente, após a avaliação dos critérios de exclusão, apenas três estudos foram considerados para análise.

**Conclusões:**

Crianças com doença hepática em preparação para o transplante podem apresentar defeitos de esmalte, pigmentação dentária, cárie, gengivite além de infecções oportunistas como a candidíase.

## INTRODUCTION

Liver transplantation is the option for patients with end-stage liver disease from diverse causes, such as:

1. Extrahepatic cholestasis;
2. Intrahepatic cholestasis;
3. Metabolic disorders;
4. Acute liver failure; and
5. Primary liver malignancy.

Infant liver transplantation has improved greatly in the last 40 years.^1,2^ This success is due to advances in surgical techniques, superior organ preservation methods, availability of more effective immunosuppressive agents, and broad accumulating worldwide experience.^3^

Liver transplantation involves three moments: pre-, trans-, and post-transplantation, all with a multidisciplinary team working to ensure minimal complications throughout the process. These teams evaluate systemic,^1,3^ psychological,^4^ and oral aspects.^4–6^ Maintaining the oral health of pediatric liver transplant recipients^7^ is important in the dental field to diagnose, treat, and prevent mouth infections both pre- and post-transplantation.^6,8^

Certain oral findings in pediatric patients with liver disease have been described in the literature, such as greenish teeth, dental hypoplasia, gingival hyperplasia, greenish gum, in addition to gingivitis and caries related to poor oral health conditions.^7,9^ These oral findings may vary depending on the moment – before or after transplantation –, and the type of liver disease.

Furthermore, the presence of opportunistic oral infections as a consequence to low immune system may arise and be associated conjointly with the underlying disease itself and the patient’s clinical condition.^8,10^ Prior to liver transplantation, dental care is essential to avoid infections of dental and/or gum origin, providing better prognosis of the transplant and improving the oral health-related quality of life of transplant patients.^8^ Although it is known that good oral condition favors a better prognosis after liver transplantation, there is still a scarcity of studies on children’s oral conditions that may impact the course of transplantation, justifying the compilation of existing data in this scoping review.

The aim of this study was to conduct a scoping review aimed to identify the oral findings/characteristics in children with liver disease in preparation for liver transplantation.

## METHOD

This scoping review is being reported according to the Preferred Reporting Items for Systematic reviews and Meta-Analyses extension for Scoping Reviews (PRISMA-ScR).^11^ The methodological framework and recommendations for this type of review are adopted by Arksey and O’Malley^12^ and the Joanna Briggs Institute.^13^ This review is registered in the Open Science Framework (OSF) (https://osf.io/) under the registration number 10.17605/OSF.IO/QCU4W.

It was not possible to execute the initial proposal regarding the exclusion criteria for studies in which the post-transplant phase was also evaluated, since articles under this format contained important information, therefore, a deviation from the protocol registered in the OSF platform for this scoping review was performed.

The research question of this review was: “What are the possible oral findings/characteristics in children with liver disease in preparation for liver transplantation?”, created based on the Population, Concept and Context (PCC) research strategy for a scoping review, in which:

1. P (population): children with liver disease considering the age group according to the World Health Organization (WHO).
2. C (concept): oral findings/characteristics.
3. C (context): in preparation for liver transplantation.

Studies that fulfilled the following inclusion criteria were considered eligible: clinical studies evaluating children with liver disease in preparation for liver transplantation; systematic reviews; prospective clinical trials (parallel group or crossover designs); observational studies (cohort, case-control, and cross-sectional); clinical case series and case reports.

Those studies that presented at least one of the following characteristics were excluded: mixed data with post-transplantation assessment; evaluation of liver transplantation together with other solid organs.

Guidelines, editorials or letters to the editor were not considered for this study.

No time limit was placed on the search, and language of publication was not restricted in the inclusion of articles.

For the search to be exhaustive, and in order to identify potentially eligible studies, different databases were considered: Medline/PubMed, Scopus and Web of Science. In addition, a gray literature search was performed through ProQuest, allowing an accurate identification of relevant information unidentified during the database search. The references of the included papers were also carefully reviewed so that articles of interest not identified by searches in the database could be considered. Since the scoping review is exploratory in nature, all results found on the topic should be included to allow researchers to identify gaps or the path being taken in the existing literature.

The search strategy was appropriately tailored for each database, following their specific syntax rules. The terms used derived from a controlled vocabulary, synonyms, related words and free terms referring to: liver transplantation; children; oral health, in combination with the Boolean operators “OR” and “AND” to allow a systematic search in the Title/Abstract field. Therefore, the search strategy established for the PubMed/Medline database was: (“child, preschool”[MeSH Terms] OR “children”[Text Word] OR “infant”[Text Word] OR “child”[Text Word] OR “toddler”[Text Word] OR “pediatric patient”[Text Word] OR “childhood”[Text Word]) AND (“liver transplantation”[MeSH Terms] OR “pediatric liver transplantation”[Text Word] OR “liver transplant”[Text Word] OR “Liver grafts”[Text Word] OR “liver disease”[Text Word])) AND (“Dental Care”[MeSH Terms] OR “Dental”[Text Word] “Pediatric Dentistry”[MeSH Terms] OR “dentistry”[Text Word] OR “Oral Health”[Text Word]), and was adapted for the other databases (Additional file 1). All search strategies were disclosed in a transparent and reproducible manner. In addition, the report of the search results and the complete selection process of the included studies were made available in the PRISMA flowchart.

The databases search was carried out until July 2021. Subsequently, all identified references were imported into the EndNote Web software to consolidate all results into a single file, and all duplicate articles were removed according to title, abstract, author and year. After this process, the remaining articles were exported to the Rayyan software^14^ for analysis according to the eligibility criteria.

The screening, eligibility and inclusion of the studies were performed by two independent reviewers (CNS and CJLG), and in case of disagreement, a joint discussion was held with a third reviewer (PSSS) considered to be an expert in the field.

Titles and abstracts of the articles identified in the database search were assessed, and those that met the inclusion criteria previously described were considered eligible. Afterwards, only studies considered eligible were screened thoroughly, and those that met any of the exclusion criteria described above were disregarded.

Once the studies were selected, the same reviewers performed the data extraction independently in a standardized spreadsheet created in Microsoft Excel specifically for this research, and discrepancies were resolved as previously mentioned. The following data were considered important for the research and were, therefore, extracted: publication details (authors, country, year, and scientific journal); sample characteristics (number and age of participants); study methodology (design, dental procedures, techniques and/or treatments compared, criteria used to assess the outcome); and information about the main results and outcome (outcomes assessed and results evaluated). Through this extraction it was possible to perform the synthesis, data interpretation and analysis presented in this review.

For this work, a narrative synthesis was performed exclusively to describe the study details, the population characteristics, and the results of the included studies, so that it is possible to answer the question posed. Conversely, risk of bias assessment was not applied in this review.^13^

## RESULTS

**Figure 1.**
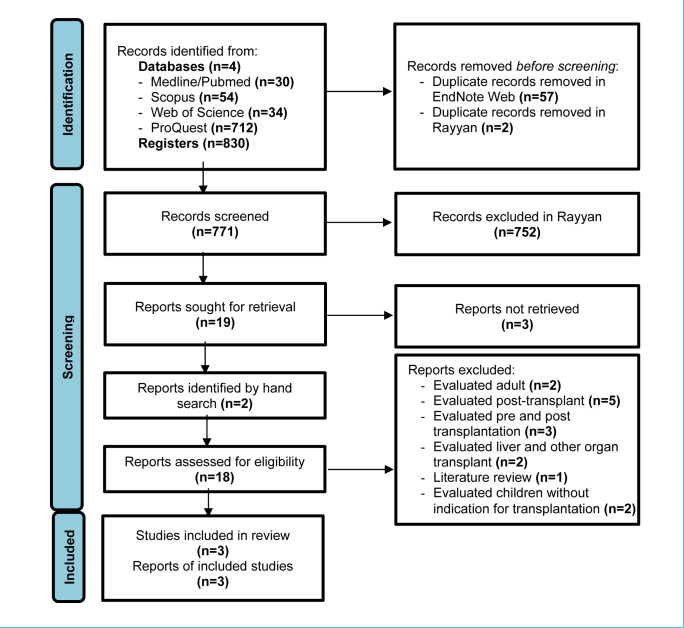
Identification of studies via databases and registers.

The primary bibliographic search generated 830 references. During the first stage, 57 articles were deleted from the EndNote Web due to duplication. While in the second stage, two articles identified as duplicates were excluded from the Rayyan software. A total of 771 articles were selected for reading by title and abstract, of which 752 were removed for not meeting the eligibility criteria based only on the initial screening. A total of 19 articles^5,9,10,15,16,17,18,19,20,21,22,23,24,25,26,27,28,29,30^ were read in their entirety. Of these, 16 were excluded because two^18,19^ evaluated oral findings/characteristics in adults; five^23,26,28,29^ reported oral features/findings/characteristics of post liver transplantation; three^9,16,28^ presented merged data of oral characteristics/findings between pre- and post-transplantation phases (which made it impossible to extract data only from the pre-transplantation phase); two^10,27^ evaluated oral conditions in two groups, liver and kidney diseases; one^16^ was a literature review; and three^21,22,24^ were excluded since the archives were not found. Later, two additional articles^31,32^ were identified through manual search of the reference lists and were fully read, but were not included once they did not meet the inclusion criteria. Finally, three studies^5,15,25^ were included in this analysis. The inclusion process of the studies can be seen in the flowchart^33^ presented in Figure 1.

All three included studies^5,15,25^ were classified as cross-sectional and involved three different countries: Poland, England, and Brazil. Furthermore, all the studies assessed children with liver diseases (Table 1) and their oral conditions (Table 2).

**Table 1.** Liver diseases evaluated in each included study.

Authors	Liver diseases
Olczak-Kowalczyk et al.^25^	- Liver cirrhosis
Sheehy et al.^15^	- Biliary atresia - Familial intrahepatic cholestasis - α-1-antitrypsin deficiency - Crigler-Najjar Syndrome - Cystic fibrosis - Hepatoblastoma - Wilson’s disease
Vidigal et al.^5^	- Biliary atresia - Familial intrahepatic cholestasis - Fulminant hepatitis - Primary sclerosing cholangitis - Cryptogenic cirrhosis - Neonatal hepatitis

**Table 2.** Oral conditions found in the studies.

Sample characteristics					Oral findings			
Authors	Country	Year	Study method	Number	Age of participants	Dental	Oral mucosa	Others
Olczak-Kowalczyk et al.^25^	Poland	2014	Cross-sectional	35	10.7±4.74 years	- Enamel defects	- Gingivitis - Mucosal lesions (Oral candidiasis) - Angular cheilitis - Cheilitis with erosions or fissures and diffuse mucosal - Overgrowth lesions of oral mucosa on the palate or/and gingivae - Erythema (atrophic stomatitis) - Pallor	-Petechiae/bleeding - Telangiectasia - Coated tongue - Black tongue - Geographic tongue -Atrophic tongue - Fissured tongue -Injure erosion on tongue margin- Lichenoid lesions in buccal mucosa; -Herpes simplex
Sheehy et al.^15^	England	2000	Cross-sectional	27	7.1±3.7 years	- Caries lesions	- Gingivitis	-NR*
Vidigal et al.^5^	Brazil	2020	Cross-sectional	60	19.0±7.44 months	- Untreated caries lesions - Dental pigmentation -Enamel defects (opacities and hypoplasia)	- Gingivitis - Dried lips - Swollen lips - Fissured lips	-NR*

NR*: not reported.

The first included study^25^ evaluated the prevalence of oral lesions in children affected by liver cirrhosis, but did not specify the type of disease. Although many features were reported, only oral candidiasis was statistically significant.

In the second study^15^, four criteria were assessed:

1. Dental caries score;
2. Plaque and gingivitis;
3. Gingival overgrowth; and
4. Oral mucosal lesions.

The evaluation was performed at two time points, before and after transplantation, but in this scoping review only the results previous to transplantation were considered, as the pre- and post-transplant data were separate. Topics (2) plaque and gingivitis, and (4) oral mucosal lesions were not statistically significant in both moments.

Finally, the last study^5^ examined the oral cavity seeking gingival inflammation, plaque, caries, developmental defects of enamel, tooth discoloration, and oral mucosal/lip alterations assessing oral health-related quality of life in pediatric liver transplant candidates.

## DISCUSSION

Oral conditions can be influenced by socioeconomic, environmental, genetic, and/or systemic factors.^34,35^ However, liver disease may have specific oral conditions. Such information becomes essential for dentists, physicians, nurses, and parents to recognize these conditions as consequences of the child’s underlying disease. At the same time, it is important to remember that preparation for transplantation undergoes some stages in which several medical specialties are involved throughout the process, including pediatric dentistry. Consequently, liver transplant patients require specialized dental care.^36^

The group of chronic liver diseases that affect children and culminate in liver transplantation can be divided according to their etiology, such as autoimmune liver diseases, metabolic diseases, vascular disorders, hepatobiliary and cryptogenic diseases.^37^ Thus, when the included studies were analyzed, we observed that the first one^25^ did not report the diseases individually. However, all liver diseases within the included studies culminated in liver cirrhosis resulting in liver transplantation. On the other hand, biliary atresia and familial intrahepatic cholestasis appeared in both other studies.^5,15^ Biliary atresia affects neonates and is the leading diagnosis in approximately 30–50% of pediatric transplant recipients^1^, which justifies the presence of young patients in the third study.^5^ All the diseases mentioned in this scoping review culminated in liver transplantation because it is the gold standard treatment for children with end-stage liver disease and metabolic disorders of hepatic origin.^3^

Regarding oral conditions, gingivitis appeared in all the studies,^5,15,25^ which may be a consequence of poor oral hygiene in children. So, programming is essential to eliminate oral sources of infection in children with liver transplantation.^15^ When dental treatment is necessary prior to transplantation, some factors should be taken into account so as to decide the timing of treatment, and whether antibiotic prophylaxis is necessary (in case of congenital heart disease or any other factor that may indicate it). These factors may be, for example, overall health and immune system condition, and hepatic dysfunction degree.^36^ For this reason, the treatment for deep caries lesion with pulpal involvement should be extraction rather than endodontics, due to the fact that it is considered definitive.^6^ However, oral surgery for such cases can lead to massive bleeding during or after surgery, hence the need for local hemostatic maneuvers^6^ together with a careful decision on postoperative medication and its dosage.

Overall, these studies^5,25^ showed that children presented dental developmental disturbances such as enamel hypoplasia, as well as lesions of the oral mucosa, with oral candidiasis. In this manner, the first finding was related to hyperbilirubinemia as a consequence of the liver disease. The second finding was related to the immunosuppression that may occur prior to transplantation due to the underlying disease itself and the drugs used to control it, such as corticosteroids and antibiotics. Moreover, these patients presented a considerable systemic imbalance.

Furthermore, the third study^5^ evaluated oral health-related quality of life. They concluded that there was a negative impact on the oral health-related quality of life of pediatric candidates for liver transplantation in relation to tooth discoloration and untreated caries lesions,^4^ based on the responses of the children’s parents.

Despite the small number of articles included in this scoping review, dental findings were identified in hepatopathic children that are also found in normoreactive children, such as dental caries. However, the treatment approach may be different in both groups. Although the topic “dental management in hepatopathic children” was not addressed in the studies, it is important to remember the peculiarities involved in liver disease during dental care in this group. Therefore, we encourage the dental scientific community to do more research on the subject. Likewise, we strongly suggest interdisciplinarity between pediatric dentistry and liver transplant medical personnel.

This scoping review is the first study to present all published reports concerning oral conditions in children previous to liver transplantation. Therefore, the limitations of our study include: the limited number of studies addressing the oral conditions and features among children prior to liver transplantation; the variety of liver diseases included in the studies; the age difference of children within the study, as well as the absence of risk of bias assessment. There is a need for studies to evaluate the relationship between oral infectious foci and its impact on systemic infections after the initiation of immunosuppressive therapy in liver transplantation. This should be the main investigation for understanding the relevance of the dentist’s action in pre- transplantation.

In conclusion, enamel defects and dental pigmentation are present in patients with liver diseases. That characteristic is related to the high rates of bilirubinemia common in these cases. In relation to the oral mucosal characteristics, opportunistic infections such as oral candidiasis are expected. And finally, in relation to the oral hygiene conditions, the presence of caries and gingivitis has also been described.

## Data Availability

The database that originated the article is available with the corresponding author.
